# Investigation of the vitamin D nutritional status in women with gestational diabetes mellitus in Beijing

**DOI:** 10.1186/s12944-017-0412-y

**Published:** 2017-01-28

**Authors:** Yanping Liu, Qiaer Jin, Yuanyuan Bao, Shanshan Li, Jing Wang, Ling Qiu

**Affiliations:** 1 Department of Nutrition, Peking Union Medical College Hospital, Chinese Academy of Medical Science, No. 1 Shuaifuyuan, Wangfujing, Dongcheng District, 100730 Beijing, China; 2 Food policy and applied nutrition/Public Health, School of Medicine, Tufts University, Medford, USA; 3 Laboratory Medicine, Peking Union Medical College Hospital, Chinese Academy of Medical Science, Beijing, China

**Keywords:** Gestational diabetes mellitus, 25-OH VitD, 25-OH VitD2, Pregnancy, Vitamin D deficiency

## Abstract

**Background:**

Vitamin D deficiency is a common issue, which has relation with GDM, during the pregnant period. To study the Vitamin D nutritional status of pregnant women with gestational diabetes mellitus (GDM) in the middle and late pregnancy and analyze the different sources of Vitamin D intake.

**Methods:**

A total of ninety-eight pregnant women with GDM were enrolled voluntarily in this study. The patients were divided into two groups, Vitamin D supplement intake and control group. The level of 25-hydroxy Vitamin D (25-OH Vit D) and the sources of Vitamin D intake and the frequency of food consumption rich in Vitamin D were investigated.

**Results:**

The incidence rate of Vitamin D deficiency (<50 nmol/L) was 20.4%. The range of serum 25-OH Vitamin D2 level was 0–24.71 nmol/L, with the detection rate of 19.4% (19/98). Eighty-four cases (85.7%) took Vitamin D supplements with duration of 2w-31w, and with average daily intake dose of 517.5 ± 113.1 IU. Patients who took Vitamin D supplements had higher serum level of 25-OH Vitamin D than who did not (74.35 ± 26.13 vs 60.45 ± 23.63 nmol/L, *p* = 0.031), and the rates of deficiency were 17.9% and 35.7%, respectively. In terms of seasonal difference, during autumn, the serum 25-OH Vitamin D2 level in the group who took Vitamin D supplements was significantly higher than control group (78.59 ± 27.54 vs 46.18 ± 18.77 nmol/L, *p* = 0.045). The diet records showed that the frequencies of consumption of dairy products and eggs among patients were 7.5 ± 3.8/week and 5.6 ± 2.2/week, respectively.

**Conclusion:**

Most of the patients took Vitamin D supplements which may help to maintain the nutritional balance of Vitamin D.

## Background

Vitamin D promotes calcium absorption in the gut and maintains adequate serum calcium and phosphate concentrations to ensure normal mineralization of the bone. It is also needed for bone growth and bone remodeling by osteoblasts. Without sufficient Vitamin D, bones can become thin and brittle. Vitamin D deficiency has been linked to rickets in children and osteomalacia in adults [1]. There are two major forms of active Vitamin D, Vitamin D2 (ergocalciferol) and Vitamin D3 (cholecalciferol). The main food sources of Vitamin D3 are dairy, egg, fish and meat. Vitamin D3 also comes from endogenous sources where it can be synthesized by skin when exposed to ultraviolet irradiation. In that reaction, the cutaneous precursor of Vitamin D, 7-dehydrocholesterol undergoes photochemical cleavage of the carbon bond between carbons 9 and 10 of the steroid ring to become active Vitamin D3. Vitamin D2 cannot be synthesized endogenously, i.e., it is an exogenous only Vitamin D, which comes from artificially synthesized compounds or some plant foods such as edible mushroom grown in natural environment. Study has shown that exposing edible mushroom to ultraviolet B light could increase its Vitamin D2 content and raise serum 25-OH VitD2 in healthy adults [2, 3]. Study also has shown that serum 25-OH VitD2 detection rate is less than 5% in China [4]. According to USDA nutrition data, the main food sources of Vitamin D are cheese (7.4 μg/100 g), egg yolk (5.4 μg/100 g), canned sardine (4.8 μg/100 g), butter (1.4 μg/100 g), and animal organs and meat products (less than 1%) [5].

Serum 25-OH Vitamin D level is recognized as the best indicator of Vitamin D status. Adequate Vitamin D status (>30 nmol/L) significantly promotes calcium absorption and reduces rickets risks, which was deemed as good Vitamin D nutritional status. Some researchers defined serum 25-OH Vitamin D lower than 50 nmol/L as diagnostic criteria for Vitamin D deficiency, 50–75 nmol/L as inadequate, and higher than 75 nmol/L as adequate [4, 6, 7].

Previously, ELISA was used to measure serum 25-OH Vitamin D level. Recently, LC-MS/MS became the gold standard of measuring Vitamin D level due to its ability to differentiate and accurately quantify the two subgroups of 25-OH Vitamin D (25-OH Vitamin D2 and 25-OH Vitamin D3); (2) high specificity, which allows differentiation of the target molecule from other metabolic intermediates, as well as avoiding interference as seen in immunological methods; (3) high sensitivity, which allows detection at ng/mL level. Such method allows nutrition researchers to identify Vitamin D food sources and evaluate dietary pattern.

Vitamin D deficiency is closely associated with Gestational Diabetes Mellitus (GDM). Vitamin D induces insulin receptor expression through Vitamin D receptor (VDR), enhancing insulin-dependent glucose transport. Vitamin D is also a potential immunosuppressant, which down-regulates the expression of pro-inflammation markers, such as TNF-α and IL-2, among pregnant women with GDM [8]. There is an increased risk of Vitamin D deficiency during pregnancy [9]. Study has found that the prevalence of Vitamin D deficiency during pregnancy is 66–96% [7]. Currently, it is recommended to use multiVitamin or calcium supplements containing Vitamin D for women during pregnancy to increase their Vitamin D intake. The dosage is based on dietary intake recommendations [10, 11]. It is important to pay attention to Vitamin D nutritional status in GDM pregnancy nutrition management. Using a new assessment technique, we investigated Vitamin D nutritional status among outpatients with GDM in our hospital, and analyzed Vitamin D sources and its association with dietary pattern.

## Methods

### Study objects

Pregnant women in middle and late pregnancy who were diagnosed with GDM by 75-g oral glucose tolerance test (OGTT) at the outpatient nutrition clinic in Peking Union Medical College Hospital from October 2013 to July 2015 were enrolled in this study. The OGTT diagnostic criteria for GDM was as follows: oral intake of 83 g glucose powder; the normal fasting glucose level was 5.1 mmol/L; 1 h post oral glucose intake was 10.0 mmol/L; 2 h post oral glucose intake was 8.5 mmol/L; diagnosis of GDM was reached if the glucose level of more than one time point was high than normal. Patients with medical history of bone mineral metabolic disease, liver or kidney dysfunction were excluded.

The inclusion criteria were: 1). Clear diagnosis of GDM after OGTT test; 2). At middle or late term of pregnancy; 3). No current medical illness; 4). Voluntary participation of the study.

The exclusion criteria are: 1). Medical history of bone mineral metabolic disease; 2). Medical history of liver disease; 3). Medical history of kidney dysfunction.

### Groups

The exogenous Vitamin D came from Vitamin D supplement or Vitamin D rich food. Patients were divided into two groups, Vitamin D supplement intake group (intervention) or control group, based on whether Vitamin D supplement was take or not before blood test. Participants took a survey about their Vitamin D supplement intake history, including brand, gestational week when starting the intake, frequency and dose, and calculation of non-dietary Vitamin D daily intake. Average daily intake of dairy products, egg, animal organ and seafood was calculated based on participants’ dietary record.

### Measurement of serum 25-OH Vitamin D

Blood was collected after overnight fasting and serum 25-OH Vitamin D2 (Molecular weight 412.66) and D3 (Molecular weight 400.63) levels were measured by using LC-MS/MS (Waters UPLC, AB Sciex 4000QTRP), and then total 25-OH Vitamin D level was calculated. According to the characteristics of the weather in Beijing, the season was divided to winter (November – February), spring and fall (March – May, September – October) and summer (June – August), the seasonal distribution of the data was also analyzed.

### Statistical analysis

Statistical analysis was performed using SPSS11.5 software. Measurement data were presented as mean ± for standard deviation. Student’s *t* test was used to compare the difference in serum 25-OH Vitamin D between the two groups. The relationship between non-dietary Vitamin D intake dose and serum 25-OH Vitamin D level was analyzed using Pearson independence test. *p* < 0.05 was considered as statistically significant.

## Results

### General characteristics of the study subjects

Ninety-eight pregnant women with GDM in the middle and late pregnancy were enrolled in the study. As shown in Table 1, the age was 33.4 ± 4.1 years old (ranges from 24 to 46 years old), the gestational weeks were 29.0 ± 3.3 weeks (ranges from 14 to 39 weeks), pre-pregnancy BMI was 23.0 ± 3.7 (ranges from 15.6 to 37.2) kg/m^2^. Sixty-eight cases were within normal BMI (BMI 18.5–23.9 kg/m^2^), eighteen cases were overweight (BMI24 ~ 27.9 kg/m^2^), five cases were obese (BMI>/=28 kg/m^2^), and seven cases were underweight (BMI < 18.5 kg/m^2^). All participants have normal liver and kidney function. There was no significant difference in age, gestational week or pre-pregnancy BMI between participants in intervention group and control group.

**Table 1:** General characteristics of study subjects

Groups	N	Age (Year)	Gestational weeks (Week)	Pre-pregnancy BMI (kg/m^2^)
Control	14	34.3 ± 4.9	28.9 ± 4.1	24.7 ± 4.5
Intervention	84	33.3 ± 3.8	28.9 ± 3.3	22.8 ± 3.5

### Comparison of serum 25-OH Vitamin D level in women of the two groups

As shown in Table 2, the serum levels of total 25-OH Vitamin D in the intervention and control group were 74. 35 ± 26.13 nmol/L and 60.45 ± 23.63 nmol/L, respectively, and the total 25-OH Vitamin D level in intervention group was significantly higher than that of the control group (*p* < 0.05). The serum level of 25-OH Vitamin D2 in the intervention group was 11.61 ± 6.71 (6.06–24.71) nmol/L, however, the serum 25-OH Vitamin D2 level in the fourteen cases in the control group was too low to be detected with the current technology (Table 2). Of the total ninety-eight patient,, there were twenty patients whose serum levels of total 25-OH Vitamin D was lower than 50 nmol/L which made them meet the criteria for the diagnosis of Vitamin De deficiency, five of them were from control group, and fifteen were from intervention group.

**Table 2:** Serum 25-OH Vitamin D levels in intervention and control groups

	25-OH VitD2 (nmol/L)	25-OH VitD2 detection rate	25-OH VitD3 (nmol/L)	Total25-OH VitD (nmol/L)	Deficiency rate (%)
Intervention (*n* = 84)	11.61 ± 6.71	22.6%	71.77 ± 26.78	74.35 ± 26.13*	17.9%
Control (*n* = 14)	0	0	0	60.45 ± 23.63	35.7%

Note: *Compared with control group, *p* < 0.05

### Seasonal changes of 25-OH Vitamin D in pregnant women with GDM

**Fig. 1:**
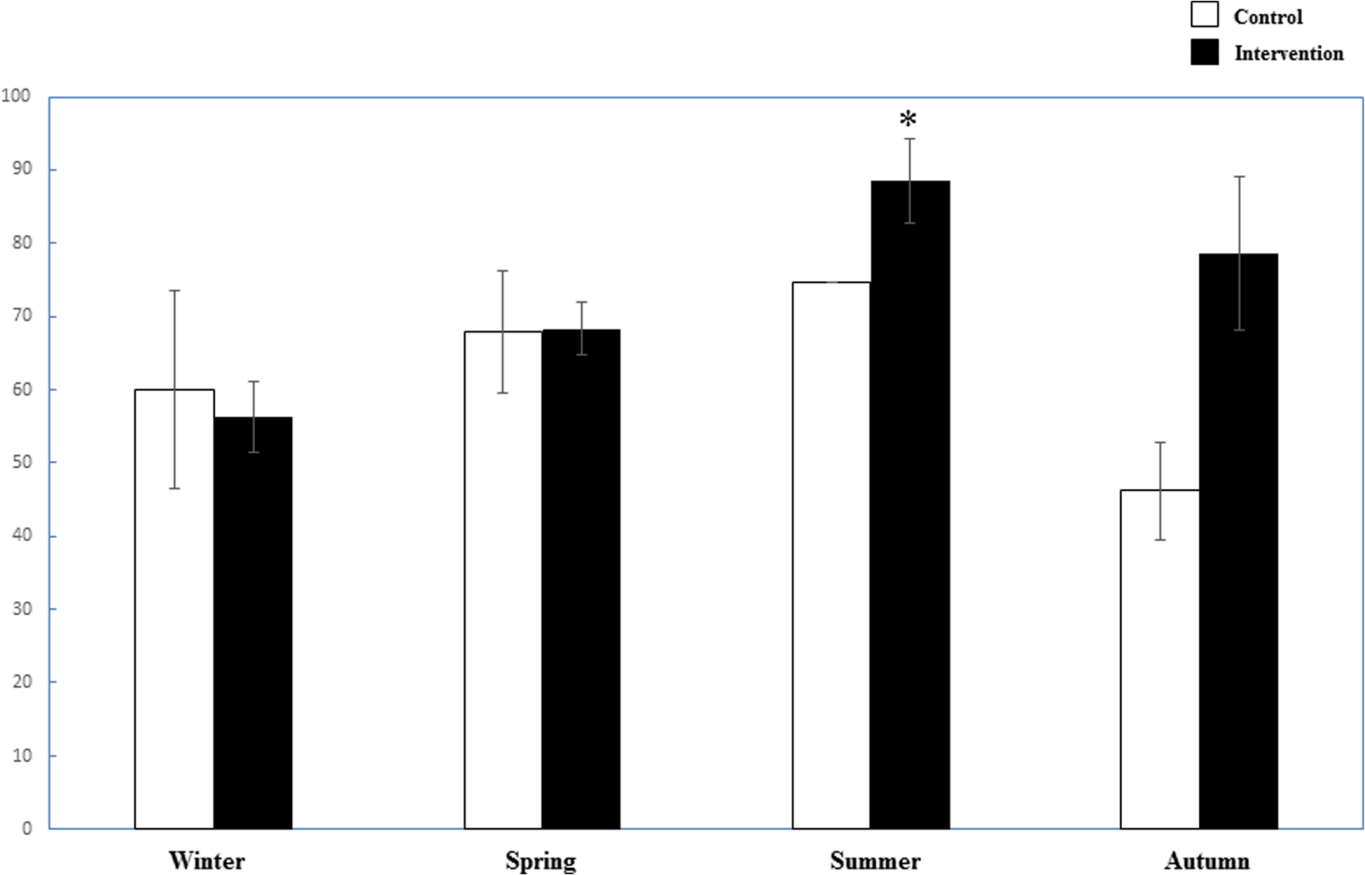
Comparison of Vitamin D deficiency rate of different seasons between the two groups. Control **□** group; **■** Intervention group. *: *p* < 0.05 compared between the two groups

Blood samples were taken between October 2013 and July 2015, twenty-one cases were collected in winter, forty-three cases in Spring, twenty two cases in Summer, and twelve cases in Autumn. Among the four seasons, serum 25-OH Vitamin D level is highest in Summer, lowest in the Winter, which is significantly lower than summer (*p* < 0.0001) and spring (*p* = 0.003). The autumn serum 25-OH Vitamin D level in the intervention group is significantly higher than that of control group. The serum 25-OH Vitamin D deficiency rate of Summer is zero in both groups, but is high of other three seasons (Fig. 1).

### Vitamin D supplement intake situation

There were fourteen out of total ninety-eight participants (19.5%) who did not take Vitamin D supplement. The average daily Vitamin D intake dose in the rest eighty-four participants was 517.5 ± 113.1 IU. Twenty-four of the participants started to take Vitamin D supplement before pregnancy, and the range of the Vitamin D supplement intake duration was 2 to 131 weeks. The Vitamin D dietary supplements they took included Vitamin D3 calcium, multi-Vitamin supplement, most are in the form of Vitamin D3, only 4 participants took supplements in the form of D2 (Forceval, Vitamin D2 200 IU/pill, once daily). There is no significant association between dose of Vitamin D supplement intake, time and serum 25-OH Vitamin D level.

### Food consumption frequency of food rich in Vitamin D

Participants filled dietary records for 7–232 days, with an average of 31.7 days. The frequency of dairy consumption is 7.5 ± 3.8 times per week, and four participants did not take dairy products at all. The frequency of egg consumption is 5.6 ± 2.2 times per week and the frequency of seafood consumption is 1.8 ± 1.2 times per week, five of the participants did not take seafood products, 15 participants had seafood for more than three times per week. Twenty participants (28%) consumed animal organ, four of them consumed animal organ for more twice per week, with an average consumption frequency 0.2 ± 0.6 times per week.

## Discussion

Normal Vitamin D level is one of the nutritional management goals for women with GDM. Most women (66–96%) in their middle and late pregnancy in Beijing are Vitamin D deficient if diagnosed with the criteria of serum 25-OH Vitamin D level <50 nmol/L (ELISA method), as indicated in two studies from Department of Endocrinology and Department of Laboratory Medicine in Peking Union Medical College Hospital [6]. This is comparable with the prevalence of Vitamin D deficiency among the general population in Beijing area (LC-MS method, 11% if diagnosed with serum 25-OH Vitamin D level <25 nmol/L, or 55% if diagnosed with serum 25-OH Vitamin D level between 25 and 50 nmol/L method) [5]. The Vitamin D deficiency level identified in this study was 20.4%, which is lower than those identified in the studies discussed previously.

Vitamin D3 can be synthesized de novo. Sunlight exposure time has an important impact on Vitamin D3 level. Subjects in this study reside in an areas from 39″26’N to 41″03’N. The annual average sunlight exposure time in this area is 2000–2500 h: 160–200 h/m in winter and 200–240 h/m in spring and fall, the sunshine intensity and temperature are appropriate for outdoor activities; up to 240–280 h/m in summer with the strongest sunshine intensity (data from data.cma.cn). The seasonal variations of serum 25-OH Vitamin D level detected in these subjects are consistent with natural environmental changes in the area of residence. Thus, the strength and duration of sunlight exposure become the main factors affecting the Vitamin D level in their bodies.

Besides sunlight exposure, Vitamin D supplementation is also an important factor that affects serum 25-OH Vitamin D level among women with GDM. This study finds that 85.7% of pregnant women received supplements containing Vitamin D. Their serum 25-OH Vitamin D levels were higher than those without supplement use. This group has a lower rate of Vitamin D deficiency (17.9%), lower than that of the control group (35.7%). In addition, participants with GDM in this study were mostly urban residents with a higher socioeconomic status. They consume more eggs, dairy, fish and animal liver products and have a more balanced diet, based on dietary records.

It is noted that the detection rate of serum 25-OH Vitamin D2 in the study is 19.4%, higher than the usual 5% in the general population [3]. Vitamin D2 was not detected among participants without Vitamin D supplement use in this study. However, only four out of nineteen participants with supplement use who was also positive for Vitamin D2 received Vitamin D2-containing supplements. 25-OH Vitamin D2 is also detected in the serum of these four participants. Therefore, consumption of Vitamin D2-containing supplements is associated with higher Vitamin D2 detection rate among pregnant women. However, other contributing factors could not be excluded.

The dietary source of Vitamin D2 can be plants and/or fortified food products. Previous studies identify edible fungi as the primary plant-based dietary source of Vitamin D. However, edible fungi were hardly seen in the dietary records among participants in this study. Such low consumption rate and amount cannot be the leading cause of high Vitamin D detection rate in these participants. Accumulation of Vitamin D2 in food chain, such as from poultry fed with Vitamin D2 fortified feeds and their eggs, also contribute to elevated Vitamin D2 levels in human bodies. Each adult egg-laying hen consumes 100–115 g/day feeds fortified with 1600 IU/kg Vitamin D (both Vitamin D2 and Vitamin D3), as indicated by Chinese nutrition standards for feeding egg-laying hens. As a result, higher egg consumption among participants in this study may explain the higher detection rate of Vitamin D2. In addition, researchers find dairy products in China are mainly fortified with Vitamin D3, which will not translate into higher Vitamin D2 detection rate among participants.

## Conclusions

In summary, 20.4% pregnant women with GDM in their middle and late pregnancy are deficient in Vitamin D, though their Vitamin D levels were higher than the general population. It is necessary to increase exogenous Vitamin D intake in this population especially in fall and winter, when there is an increase of Vitamin D deficiency in Beijing area. This group often uses dietary supplements containing Vitamin D. They also have a Vitamin D2 detection rate of 19.4%, which can be explained by consuming edible fungi or supplements containing Vitamin D2. Alternatively, higher eggs and poultry consumption may also explain the high detection rate. Further study is needed to confirm this.

## Abbreviations

GDM: Gestational diabetes mellitus
OGTT: Oral glucose tolerance test
25-OH Vit D: 25-hydroxy Vitamin D
